# Histopathological Determinants of Survival in Resected Cases of Pancreas Cancer

**DOI:** 10.1155/1993/83609

**Published:** 1993

**Authors:** S. T. Brower, R. M. Newman, D. Pertsemlidis, I. Kreel, A. H. Aufses

**Affiliations:** 1 Department of Surgery Division of Surgical Oncology Mount Sinai Medical Center New York New York USA; 2 Division of Surgical Oncology Mount Sinai Medical Center Dept of Surgery- Box 1259 1 Gustave L. Levy Place New York New York 10029 USA

## Abstract

We have examined the histopathological factors affecting the degree of local spread, regional lymph node (RLN) metastases, and overall survival (O.S.) in a group of 39 cases of resected carcinoma of the exocrine pancreas. Although the mean O.S. for the group was 14.3 months, resected patients without RLN involvement had a mean survival of 24 months. In contrast the mean O.S. rate was 8 months for patients with RLNs involved. Size, tumor location, and histological grade were compared to RLN involvement and O.S. The mean size of primary tumor did not differ significantly between patients with or without RLN's (r.1 versus 4.6cms). However, 7 or 8 T_1_ tumors were <4cm and 35% of tumors <4cm were T_1_ lesions. In contrast, only of 17 tumors (6%) >4cm was T_1_. Histological grade was correlated with nodal status and O.S. There was a significant difference between histological grade and the presence of metastatic lymph nodes (G1, 37% positive, G2-4.50% positive). Patients with well differentiated tumors had a mean survival of 21 months compared to a mean survival of 10 months for less differentiated tumors (*p*<0.05). This difference was even more significant when stratified for nodal status. The patients with well differentiated tumors and no RLN involvement had a mean survival of 32.5 months compared to 8.6 months for well differentiated tumors with RLN involvement. In summary, we have shown that size, histological grade, and local spread predict for nodal status. However, specific patient subsets (G1, node negative) may exhibit an excellent survival when curative pancreas resection is successful.


*HPB Surgery*, 1993, Vol. 7, pp. 1-14
Reprints available directly from the publisher
Photocopying permitted by license only
© 1993 Harwood Academic Publishers GmbH
Printed in the United States of America

### HISTOPATHOLOGICAL DETERMINANTS OF SURVIVAL IN RESECTED CASES OF PANCREAS CANCER

S.T.BROWER, R.M.NEWMAN, D.PERTSEMLIDIS, I.KREEL and A.H.AUFSES, Jr.

*Department of Surgery, Division of Surgical Oncology, Mount Sinai Medical Center, New York, New York, USA*

We have examined the histopathological factors affecting the degree of local spread, regional lymph node (RLN) metastases, and overall survival (O.S.) in a group of 39 cases of resected carcinoma of the exocrine pancreas. Although the mean O.S. for the group was 14.3 months, resected patients without RLN involvement had a mean survival of 24 months. In contrast the mean O.S. rate was 8 months for patients with RLNs involved. Size, tumor location, and histological grade were compared to RLN involvement and O.S. The mean size of primary tumor did not differ significantly between patients with or without RLN's (r.1 versus 4.6cms). However, 7 or 8 $T_1$ tumors were <4cm and 35% of tumors <4cm were $T_1$ lesions. In contrast, only 1 of 17 tumors (6%) >4cm was $T_1$. Histological grade was correlated with nodal status and O.S. There was a significant difference between histological grade and the presence of metastatic lymph nodes (G1, 37% positive, G2-4.50% positive). Patients with well differentiated tumors had a mean survival of 21 months compared to a mean survival of 10 months for less differentiated tumors ($p < 0.05$). This difference was even more significant when stratified for nodal status. The patients with well differentiated tumors and no RLN involvement had a mean survival of 32.5 months compared to 8.6 months for well differentiated tumors with RLN involvement. In summary, we have shown that size, histological grade, and local spread predict for nodal status. However, specific patient subsets (G1, node negative) may exhibit an excellent survival when curative pancreas resection is successful.

KEY WORDS: Pancreas cancer, histopathology, survival

### INTRODUCTION

Pancreas cancer is the fourth most common cause of cancer related mortality in the United States[1-2], with an overall survival rate of 0-5%. Despite advances in diagnostic technology, most patients are identified by clinical presentation, and associated local and distant spread[2-5]. Even in patients wtih localized disease, mean survival remains less than 16 months after attempted curative resection[3,6-7]. Surgical resection, however, remains the only chance for long term survival[3,6-7].

Certain pathological characteristics of the pancreatic cancer seem to be important in predicting outcome[8-10]. The degree of local spread of the primary tumor and status of the regional lymph nodes have been recognized as important determinants of staging by the American Joint Committee on Cancer[1-2]. By understanding how characteristics of pancreatic malignancies effect the degree of local spread and

This work is supported by an American Cancer Society Grant #88-125(STB)

Address correspondence to: Steven T. Brower, MD, Assistant Professor of Surgery, Chief, Division of Surgical Oncology, Mount Sinai Medical Center, Dept of Surgery - Box 1259, 1 Gustave L. Levy Place, New York, New York 10029, USA

nodal involvement, subsets of patients may be identified that would be more amenable to attempted curative resection.

We retrospectively reviewed pathological data from resected cases of pancreatic cancer with particular attention to the extent of loco-regional invasion, histopathological characteristics of the primary tumor, lymphatic invasion, size, and, differentiation. We have attempted to analyze those factors that may have influenced postoperative survival.

### MATERIALS AND METHODS

Fifty-four cases of attempted curative resection for pancreatic neoplasms were retrospectively reviewed. Surgery for these patients was performed at The Mount Sinai Medical Center from 1985 to 1990. The pathological material from all patients was reviewed and confirmed the diagnosis of malignant pancreatic neoplasm (duct cell adenocarcinoma $n=37$). Islet cell tumors (11 cases) and mucinous-cystadenocarcinomas (6 cases) were excluded). The study group consisted of 54% males and 46% females, with an average age at operation of 63 years.

American Joint Committee on Cancer (AJCC) staging criteria were used to categorize patients into TNM stages as follows; Degree of local spread is delineated by: $T_1$; tumors limited to the pancreas, $T_2$; tumors directly spread to peripancreatic tissue, bile duct, or duodenum, and $T_3$; tumors directly spread to the stomach, colon, spleen, or adjacent large vessels. Regional Lymph node status is categorized by: NO; nodes uninvolved and N1; nodes involved[7]. Size refers to the largest dimension of the primary tumor as reported by surgical pathology. Histologic grade of the primary tumor was divided into two groups; well differentiated (gl), and less well differentiated (G2-G4) including moderately, poorly, and undifferentiated tumors.

The types of surgery performed included pancreaticoduodenectomies (Whipple procedure $n=22$), total pancreatectomies ($n=11$), and distal pancreatectomies ($n=4$). Although clinically involved as well as clinically uninvolved peripancreatic and regional nodes were included in the resections, no attempt was made to perform a radical regional lymphadenectomy as advocated by other authors[7,11-12]. An average of 9.5 major pancreatic resections (pancreaticoduodenectomy and total pancreatectomy) were performed each year. Although the procedures were performed by a number of surgeons, the maximum average number of major resections performed by an individual surgeon was 4.5 in each year. The performance of total pancreatectomy followed a bell-shaped distribution over the five year period with the maximum number of 4 performed per year being reduced to 2 since 1988.

Adjuvant chemotherapy and radiation therapy were administered according to various experimental protocols. Although no standardized treatment was universally administered, the total number of patients receiving adjuvant chemotherapy after surgery was 11. Five patients received regional radiation therapy after attempted curative resection.

Actuarial survival was calculated using Kaplan-Meier plots[13]. A multivariate Cox proportional hazards model was used to determine which independent factors jointly predicted long term survival[14].

### RESULTS

### *Areas of Local Spread in Relation to Position of Primary Tumor*

Of the thirty seven patients, eight had tumors limited to the pancreas ($T_1$), twenty-one were designated $T_2$, and eight had other organ involvement or large vessel encasement ($T_3$). Any one primary tumor may have spread to several loco-regional areas (Table 1). The areas of local spread are logically dictated by the position of the primary tumor.

As seen in Table 1, the most direct sites of local spread were in the peripancreatic tissues, bile duct and duodenum. The incidence of peripancreatic tissue involvement was approximately equal for tumors located in the head versus the body and tail. 50% of tumors in the head of the pancreas that involved the peripancreatic tissue had no invasion of the duodenum or the bile duct.

Invasion of adjacent large vessels was seen in only three resected specimens. Interestingly, all of the resections for cure which included local spread to adjacent large vessels were tumors located in the pancreatic head. The most common sites of contiguous organ involvement included the stomach, spleen and colon. Direct invasion of the transverse colon and transverse mesocolon was noted in one each of the body and tail lesions. Three cases (8%) demonstrated direct invasion of the stomach. Similarly three lesions were found to invade the splenic hilum.

**Table 1** Areas of local spread in relation to position of primary tumor. The tumors were divided into $T_1$, $T_2$, $T_3$ spread and analyzed by position of primary tumor. See Materials and Methods section for designation of T stage

| *Degree of Spread* | *Head* | *Body* | *Tail* |
|---|---|---|---|
| T1 | | | |
| Limited to Pancreas | 7 | — | 1 |
| T2 | | | |
| Peripancreatic Tissues | 10 | 4 | 4 |
| Duodenum | 4 | — | 1 |
| Bile Duct | 7 | — | — |
| T3 | | | |
| Stomach | 1 | 1 | 1 |
| Spleen | — | 1 | 2 |
| Colon | — | 1 | 1 |
| Adjacent Large Vessels | 3 | — | — |

**Table 2** Local spread and position of primary pancreatic tumor

| | | *TNM Classification* | | |
|---|---|---|---|---|
| | | *T1* | *T2* | *T3* |
| Head | (n = 24) | 7 (24%) | 13 (54%) | 4 (17%) |
| Body | (n = 5) | 0 | 4 (80%) | 1 (20%) |
| Tail | (n = 8) | 1 (13%) | 4 (20%) | 3 (37%) |
| Total | | 8 | 21 | 8 |

Of 13 patients with $T_2$ carcinoma of the head of the pancreas, 12 tumors involved the bile duct, duodenum or both. Ten of the 13 patients had peripancreatic direct invasion of the retroperitoneum.

Table 2 demonstrates that 52% of patients had $T_2$ degree of local spread. Of the tumors limited to the pancreas alone ($T_1$), 88% of them were in the head. However, only 24% of the total lesions of the head were confined within the pancreatic capsule. In contrast, only 1 of 13 body and tail lesions was contained within the pancreas. Tumors of the tail had the largest percentage (37%) of contiguous organ spread ($T_3$).

The size of the primary tumor correlated with the percentage of the tumors that were localized. This effect of size on local spread is seen in Table 3. As one might expect, 7 of 8 (88%) $T_1$ tumors were smaller than 4cm. In contrast $T_3$ tumors tended to be larger (>4cm) with 6 of 8 (75%) tumors falling into this category. Small tumors, however, did not protect against contiguous loco-regional spread to bile duct, duodenum or peripancreatic tissues. The $T_2$ tumors were equally as likely to be less than or greater than 4 cm (55% and 59% respectively). When the data comparing size and degree of local spread were stratified for location of the primary tumor, the degree of local spread remained similar to the group as a whole (data not shown). Therefore, it seems that size, as a predictor of local spread, is similar for different regions of the pancreas.

Table 4 analyzes the relationship of histological grade and degree of local spread. Sixteen tumors were well differentiated neoplasms as compared to 21 tumors with moderately to poorly differentiated histology. The degree of differentiation was a significant factor in predicting which tumors were limited to the pancreas. 31% of the well differentiated tumors were T1 lesions whereas only 14% of the less well differentiated group were contained within the pancreas ($p < .05$). In contract, the T2 lesions demonstrated histologies that were more undifferentiated in 67% of the specimens ($p < .005$). Patients with T3 local spread were equally as likely to have well differentiated versus less well differentiated tumors. Although the well differentiated tumors were equally distributed among the different types of local spread ($T_1$, $T_2$, $T_3$), the less well differentiated pancreatic carcinomas were more often seen with peripancreatic spread.

**Table 3** The effect of size of primary tumor on degree of local spread

| | | *TNM Classification* | | |
|---|---|---|---|---|
| *Size* | *n* | *T1* | *T2* | *T3* |
| <4cm | 20 | 7 (35%) | 11 (55%) | 2 (10%) |
| >4cm | 17 | 1 (6%) | 10 (59%) | 6 (35%) |

**Table 4** Histologic grade and degree of local spread

| | | *TNM Classification* | | |
|---|---|---|---|---|
| *Histologic grade* | *n* | *T1* | *T2* | *T3* |
| Well Differentiated (G1) | 16 | 5 (31%) | 7 (44%) | 4 (25%) |
| Less Well Differentiated (Grades 2, 3 & 4) | 21 | 3 (14%) | 14 (67%) | 4 (19%) |

Table 5 examines the effect of size and grade on the degree of local spread with tumors stratified for $\leq 4$ cm versus tumors $>4$ cm). For lesions in the small tumor size group, a statistically significant difference was seen when comparing histological grade and the degree of local spread. Of the 20 patients identified with small tumors, 10 were less well differentiated. The well differentiated group had 50% of tumors limited to the pancreas at the time of resection whereas only 20% of the less well differentiated group were so localized ($p < .005$).

Among the large tumor group, only one tumor %9%0 that was less well differentiated was also confined to the substance of the pancreas. Therefore it would seem that in the larger tumor size group that the distribution for local spread seemed to follow what would be expected on the basis of size alone without regard to differentiation. This is in contrast to the smaller sized tumors. When examined for size alone 35% (7 of 20) tumors $<4$ cm were limited to the pancreas. This is contrasted to the finding of 50% of well differentiated tumors $\leq$4cm which were limited to the pancreas.

The influence of local spread on overall survival is seen in Table 6. As seen, patients with tumors limited to the pancreas demonstrated superior survival when compared to tumors with peripancreatic ($p < 0.05$) or contiguous organ spread ($p < .001$). There is an obvious trend of decreased survival with increasing degree of local spread.

This difference in survival is similarly seen in the actuarial curve of Figure 1. The differences in actuarial survival are statistically significant when $T_1$ tumors are compared to $T_2$ and $T_3$ cancers. At 24 and 36 months, the actuarial survival of $T_1$

**Table 5** The effect of size and grade on degree of local spread. The tumors were stratified for size <4cm or ≥4cm and the T stage identified. The asterisk indicates a comparison between groups with a $p$ value <0.05

| | | *TNM Classification* | | |
|---|---|---|---|---|
| *Size* | *Number* | *T1* | *T2* | *T3* |
| <4cm (n = 20) | | | | |
| Well Differentiated (G1) | 10 | 5 * | 4 | 1 |
| Less Well Differentiated (G2, 3 & 4) | 10 | 2 | 7 | 1 |
| >4cm (n = 17) | | | | |
| Well Differentiated (G1) | 6 | — | 3 | 3 |
| Less Well Differentiated (G2, 3 & 4) | 11 | 1 | 7 | 3 |

$p = 0.05$

**Table 6** Overall survival and degree of local spread. The mean overall survival was noted for three groups $T_1$, $T_2$, $T_3$

| *Degree of Spread* | *Overall Survival (months)* |
|---|---|
| T1 | 21.0 |
| T2 | 15.8 |
| T3 | 5.4 |

$p < 0.05$ for groups T1, T2; $p < 0.01$ for groups T2, T3; $p < 0.001$ for groups T1, T3

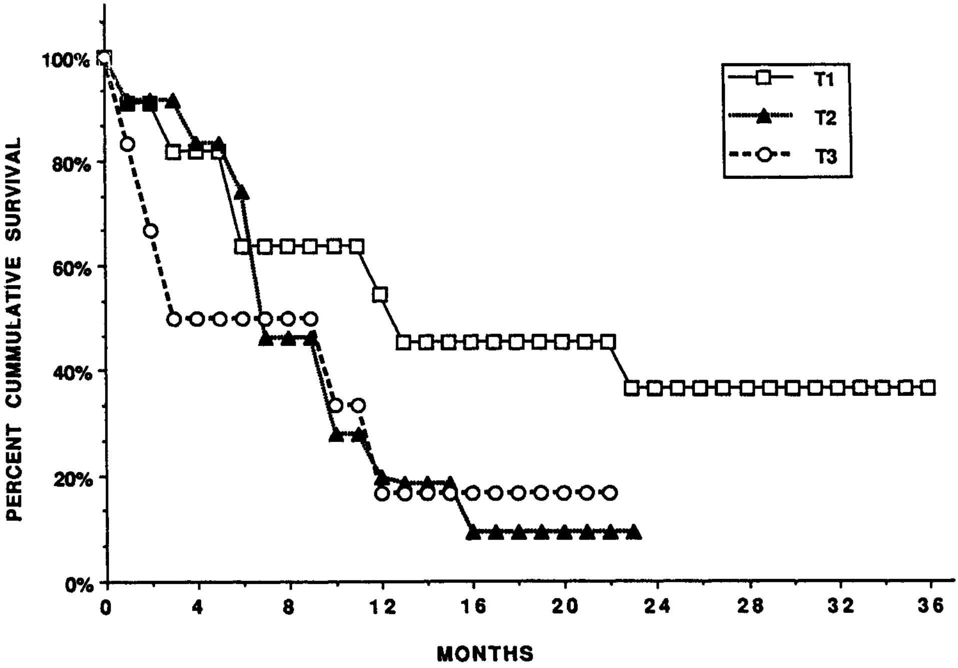

**Figure 1** Actuarial survival as a function of T stage. □ $T_1$, . . .▲ . . . $T_2$, . . .o . . . $T_3$.

patients was 31%. This is contrasted to diminished survival of 9% and 5% for $T_2$ and $T_3$ cancers.

### *Regional Lymph Node Involvement*

Of the thirty-seven patients resected for cure, pathologic data for metastatic regional lymph node involvement was available on 35 patients (Table 7). Sixteen patients (45%) were free of nodal involvement and 19 patients (55%) had at least one positive lymph node. We had previously demonstrated that the degree of local spread could influence survival following curative resection (Table 6, Figure 1). We next examined whether this effect was due to the status of regional lymph nodes. As shown in Table 7, among the various degrees of local spread, only $T_1$ tumors (limited to the pancreas) showed any significant difference in favor of negative regional lymph node status. About 2/3 of the patients with tumors confined to the pancreas were free of regional lymph node involvement. This was in sharp contrast for $T_2$ or $T_3$ cancers where 60% of the tumors were metastatic to the regional lymph nodes.

Table 8 examines the sites of local spread as a predictor for regional lymph node involvement. Other than for $T_1$ tumors limited to the pancreas in which 63% of the patients had no metastatic lymph nodes, all other sites of contiguous spread failed to differentiate between positive or negative lymph nodes. For example, the 17 patients who demonstrated peripancreatic retroperitoneal involvement were equally as likely to have uninvolved or involved regional lymph nodes. Similarly,

**Table 7** Degree of local spread and regional lymph node (RLN) status

| *TNM Tumor Classification* | | *No RLN Involvement* | | *RLNs Involved* | |
|---|---|---|---|---|---|
| T1 | (n = 8) | 5 | (63%) | 3 | (37%) |
| T2 | (n = 20) | 8 | (40%) | 12 | (60%) |
| T3 | (n = 7) | 3 | (43%) | 4 | (57%) |

$p$ <0.05 for T1 vs T2 or T3.

**Table 8** Sites of local spread and regional lymph node involvement

| *Degree of Spread* | | *Regional Lymph Nodes Not Involved* | | *Regional Lymph Nodes Involved* | | *p Value* |
|---|---|---|---|---|---|---|
| T1 | | | | | | |
| Limited to Pancreas | n = 8 | 5 | (63%) | 3 | (37%) | <0.05 |
| T2 | | | | | | |
| Peripancreatic Tissue | n = 17 | 8 | (47%) | 9 | (53%) | ns |
| Duodenum & Bile Duct | n = 12 | 5 | (42%) | 7 | (58%) | ns |
| T3 | | | | | | |
| Stomach | n = 2 | 1 | (50%) | 1 | (50%) | ns |
| Spleen | n = 2 | 1 | (50%) | 1 | (50%) | ns |
| Colon | n = 1 | 1 | (100%) | 0 | | ns |
| Adjacent Large Vessels | n = 3 | 1 | (33%) | 2 | (67%) | ns |

**Table 9** Histologic grade and nodal status

| *Grade* | *No RLN Involvement* | | *RLNs Involved* | |
|---|---|---|---|---|
| G1 | 9 | (63%) | 6 | (37%) |
| G2-4 | 13 | (50%) | 13 | (50%) |

the 12 patients demonstrating direct extension to the duodenum or bile duct, did not predict the presence of regional lymph node involvement.

As seen in Table 9, the degree of differentiation of the primary tumor was a significant determinant of nodal status in the well differentiated group. As previously stated, only $G_1$ tumors were different when examined for regional lymph node involvement (63% versus 37% respectively). The more undifferentiated tumors were equally as likely to have uninvolved or metastatic nodes present as 50% of patients with G2-4 histology were free of positive lymph nodes. Among patients with lesions of the head, an equal number had positive nodal involvement or the absence of regional lymph nodes (RLNs). Twelve of 24 patients with tumors confined to the head of the pancreas were without evidence of regional lymph nodes. For the 11 patients with tumors within the body and tail of the pancreas 7 of 11 (63%) contained positive lymph nodes.

With respect to the size of tumors and the relationship on nodal status, small tumors $\leq$4cm were just as likely to have node free disease or RLNs involved. However, for tumors $>$4 cm, RLNs were twice as likely to have microscopic involvement than to be free of disease.

*Survival*

The operative mortality for the entire group was 5.8% (2/37 patients). This figure compares favorably with other reported perioperative mortality rates for major pancreatic resection[6,8,15-17]. Ths mean overall survival for the group was 14.3 months following resection (Table 10). When the group was further stratified by the degree of local spread, $T_1$ patients had a median survival of 24.5 months. This was statistically different from the median survival of $T_2$ (8.3 months) and $T_3$ (7.8 months).

Survival as a function of regional lymph node status was next examined. As demonstrated in Table 10 the overall survival for patients with negative lymph nodes was 23.5 months. This is in contrast to the overall survival for patients with positive lymph nodes which was 7.4 months ($p < .05$). Figure 2 demonstrates the overall survival for node negative and node positive patients at 36 months. 31% of the node negative pancreatic cancer patients were alive at 36 months. This is in sharp contrast to 5% of patients alive with positive lymph nodes. The survival by nodal status was further stratified by size and histologic grade (Table 11). There remained a significant difference for mean survival between node negative and node positive patients for tumors $\leq 4$ and tumors greater than 4 cm.

The combination of differentiation and nodal status demonstrated differences in survival for node negative patients. For patients with negative regional lymph nodes, well differentiated status predicted for improved survival when compared to less well differentiated status (32.5 months versus 14.4 months, respectively $p < .05$). In contrast, the finding of positive lymph nodes was more important than differentiation status for survival. There was no difference in survival between well differentiated and less well differentiated positive node patients.

In summary, there remained a significant difference in overall survival between node negative patients and node positive patients in all groups. Most importantly, a subset of patients with well differentiated tumors and regional lymph nodes free of disease demonstrated the longest survival in this series of 32.5 months from the time of resection. This differed from a subset of patients with less well differentiated, node free disease who had a mean survival of 14.4 months from resection. No stratification of node positive patients predicted for improved survival within this poor prognosis group of patients.

**Table 10** Overall survival after pancreas resection for pancreatic carcinoma. Overall survival was analyzed as a function of both local spread and the regional lymph node status

| | | *Survival from Operation (months)* | |
|---|---|---|---|
| Overall Mean Survival | | 14.3 | |
| Local Spread | T1 | 24.5 | * (T1 vs T2); ** (T1 vs T3) |
| | T2 | 8.3 | |
| | T3 | 7.8 | |
| Regional Lymph Nodes | N0 | 23.5 | ** (N0 vs N1) |
| | N1 | 7.4 | |

*($p$ <0.05); **($p$ <0.01).

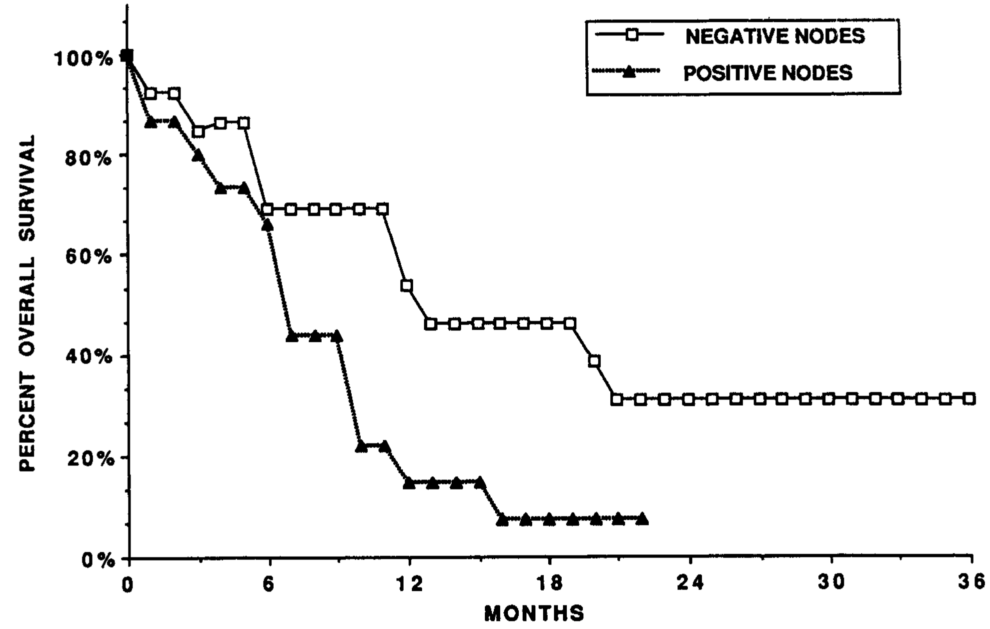

**Figure 2** Actuarial survival as a function of regional lymph node status. Negative regional lymph nodes –□–Positive regional lymph nodes . . .▲ . . .

**Table 11** Survival by regional lymph node status as a function of tumor size and grade. Tumors were stratified by size, differentiation, and nodal status and analyzed for overall survival

| | *Overall Survival (months)* | |
|---|---|---|
| *Regional Lymph Nodes* | *Negative (N0)* | *Positive (N1)* |
| *Size* | | |
| <4cm | 28.2 ⌉ ns | 5.9 ⌉ ns |
| >4cm | 15.0 ⌋ | 9.1 ⌋ |
| *Grade* | | |
| Well differentiated | 32.5 ⌉ * | 8.6 ⌉ ns |
| Less well differentiated | 14.4 ⌋ | 6.8 ⌋ |

($p$ <0.05) for all node negative vs node positive groups; *($p$ <0.05).

### DISCUSSION

A number of recent studies have demonstrated a trend for improved survival after curative resection for carcinoma of the pancreas with some institutions having reported five year actuarial survival rates of 20%[6,18-19]. In addition, a well organized prospectively randomized trial from the Gastrointestinal Tumor Study Group (GITSG) has demonstrated that combination radiotherapy and chemotherapy may double mean survival in selected patients after resection for carcinoma of the head of the pancreas[20].

Most of these studies have attempted to identify prognostic factors relevant in predicting long term survival. Many factors have been identified as important predictors of improved surgical outcome[3,7,29]. Some of these include improved technical aspects for the performance of pancreatectomy as well as regionalization and specialization for major pancreatic resection. The results of our study predict that this improvement should be interpreted within the context of clearly defined histopathological parameters of the tumor that may influence long term survival. In our series, both the degree of local spread and regional lymph node metastases had a significant effect on survival after operation.

We have shown a median overall survival for the entire group of resected cancer patients of 14.3 months and actuarial survival of 17.6% at 36 months. These results seem to compare favorably with other large series of reported cases of resectable carcinoma of the pancreas[6,21-22]. This survival is the result of a number of complex factors the most important of which is the biological aggressiveness of the tumor as measured by the pathological stage.

Other recognized important factors include delay to presentation, perioperative mortality, and other more recently recognized independent prognostic factors such as blood transfusions[6-7,17,22-23]. We have shown, however, that with careful subset analysis, groups may exist that exhibit satisfactory long term results. For example we have shown that tumors confined to the pancreas exhibit from 1 1/2 to 4 fold increase in the median overall survival when compared to locally infiltrating or regionally advanced pancreas cancer (see Table 10). More importantly the absence of regional lymph nodes confers a three-fold increased survival advantage over node positive patients, with the median overall survival for patients with negative lymph nodes approximating 21 months (see Figure 2). A smaller tumor size and negative lymph nodes does not yield an increased survival advantage over node negative tumors >4cm. Conversely, the association of a well differentiated tumor and negative lymph nodes confers a statistically significant survival advantage compared to patients with moderately or poorly differentiated cancers and negative lymph nodes (26.8 versus 16.7 months, respectively). Unfortunately, we have not recognized any subset of node positive patients in which either size, differentiation, or location of tumor confers a survival benefit.

It has been previously recognized that most early carcinomas of the pancreas are located in the pancreatic head[12,24]. In our series, 88% of the $T_1$ tumors were small tumors located in the head of the pancreas. However, 2/3 of these small pancreatic head lesions are associated with metastatic carcinoma. Furthermore, although the chance for a tumor to be localized ($T_1$) is greater in lesions in the head than for other regions, only the minority of the resected pancreatic head carcinomas in our series were limited to the pancreas at the time of operation.

Similarly, when examining $T_2$ lesions, small tumor size (<4cm) did not accurately predict confined local spread or nodal status. Large tumors >4 cm as a group were characterized by a greater degree of local spread and regional lymph node involvement. This frequent spread of small tumors is reflected by the absence of increased survival for small tumors compared to large tumors when one stratifies the patients by similar nodal status.

The degree of histologic differentiation seems to favorably effect the degree of local spread of the primary in our series. Patients with well differentiated tumors were also more likely to be free of lymph node metastases. These effects may have contributed to the reasonable survival enjoyed by the subset of patients with well differentiated node free disease demonstrated to be significantly different from a less well differentiated node free counterpart.

Our results of 31% actuarial survival at 36 months for node negative patients is similar to other recent studies reporting increased survival rates for this disease. It should be emphasized that in our series, the absence of regional lymph nodes was not a minor percentage of patients but involved 16 of 35 patients or 45% of the entire group.

A number of characteristics of the cumor were significant in predicting long term survival. Of particular note was the absence of lymph nodes, well differentiated status of the primary tumor, and degree of local spread ($T_1$ versus $T_2$, $T_3$). Size did not seem to significantly impact on overall survival. When these factors were evaluated for multivariant analysis, the presence or absence of lymph node metastases was the single most important factor in determining improved survival.

Prior studies have examined survival rates following resection of pancreas carcinoma and have quoted survival rates less than 10%. These studies have not necessarily examined subsets of patients with the required vigorous attention to histopathological detail that more recent studies and our present study have utilized. As a result of the poor results, there has been little enthusiasm for further improvement with adjuvant therapy[25]. More importantly it has not been easy to identify subsets of patients that would benefit from the possibility of radiation therapy and combination chemotherapy. We have demonstrated that patients without lymph nodes, tumor confined to the pancreas, and well differentiated status may have a survival status that would benefit from adjuvant therapy. There is little information available regarding adjuvant treatment after surgical resection for cancer of the pancreas. A study of the Gastrointestinal Tumor Study Group, although based on few patient numbers, suggested that there may be an advantage to adding 5FU plus external beam radiation in some patients with a two year survival improvement of 43% versus 18% for the control patients who received surgery alone. The results of our study and others showing improved survival should further encourage the development of new combination multidisciplinary therapies such as radiosensitization, chemohormonal synchronization, and antibody targeted direct adjuvant therapies including growth factor and signal transduction protein inhibitors for pancreas carcinoma that have been shown to have some benefit *in vitro*[26].

In conclusion, although the overall results for the majority of patients after resection for pancreas carcinoma are poor we have shown in defined subsets that a reasonable survival may be achieved. The size of the tumor is often a misleading guide to the degree of local spread or nodal status. Histologic grade of the primary tumor, substantiated by DNA synthetic rates,[27] may be more helpful in predicting localized disease and improved survival after attempted curative resection for carcinoma of the pancreas. For the node negative group of patients survival rates may be similar to other neoplasms within the gastrointestinal tract such as colon cancer who recently have benefited greatly from successful adjuvant therapy.

*Acknowledgements*

The authors wish to gratefully acknowledge Ms Joanne Madio for the excellent preparation of this manuscript and Ms Joan Bratton for the meticulous data collection and statistical analysis.

### *References*

1. Staging of Pancreas Cancer (1988) In: *Manual for Staging of Cancer*, 3rd edition. Ed: Beahrs, O.H., Henson, D.E., Hutter, R.V.P., Myers, M.H. J.B. Lippincott Co., New York, pp. 109–115
2. Pollard, H. (1981) Staging of cancer of the pancreas: cancer of the pancreas task force. *Ca*, **47**, 1631–1637
3. Gudjonsson, B., Livstone, E. and Spiro, H. (1978) Cancer of the pancreas: diagnostic accuracy and survival statistics. *Ca*, **42**, 2494–2506
4. Kalser, M., Barkin, J. and MacIntyre, J. (1985) Pancreatic cancer: assessment of prognosis by clinical presentation. *Ca*, **56**, 397–402
5. Moossa, A. (1982) Pancreatic cancer: approach to diagnosis, selection for surgery and choice of operation. *Ca*, **50**, 2689–2698
6. Cameron, J., Crist-David, W., Sitzmann, J., Hruban, R., Boitnott, J., Seidler, A. and Coleman, J. (1991) Factors Influencing Survival After Pancreaticoduodenectomy for Pancreatic Cancer. *Amer.J.of Surg.*, **161**(1), 120–125
7. Connolly, M., Dawson, P., Michelassi, F., Moossa, A. and Lowenstein, F. (1987) Survival in 1001 Patients with Carcinoma of the Pancreas. *Ann.Surg.*, **206**, 366–373
8. Mannell, A., van Heerden, J., Weiland, L. and Ilstrup, D. (1986) Factors influencing survival after resection for ductal adenocarcinoma of the pancreas. *Ann.Surg.*, **203**(4), 403–407
9. Nagai, H., Kuroda, A. and Morioka, Y. (1986) Lymphatic and local spread of T1 and T2 pancreatic cancer. A study of autopsy material. *Ann.Surg.*, **204**, 65–70
10. Poston, G., Gillespie, J. and Guillou, P. (1991) Progress Reports: Biology of pancreatic cancer. *Gut*, **32**(7), 800–812
11. Cubilla, A., Fitzgerald, P. and Fortner, J. (1978) Pancreas cancer-duct cell adenocarcinoma: survival in relation to site, size, stage, and type of therapy. *J.Surg.Oncol.*, **10**, 465–482
12. Cubilla, A., Fortner, J. and Fitzgerald, P. (1978) Lymph node involvement in carcinoma of the head of the pancreas area. *Ca*, **41**, 880–887
13. Kaplan, E. and Meier, P. (1958) Non paramentric estimation from incomplete observations. *J.of Stat.Anal.*, **53**, 457–481
14. Cox, D.R. (1972) Proportional multivariant analysis regression models and life tables. *J. of Stat.Anal.*, **34**, 187–220
15. Matsuno, S. and Sato, T. (1986) Surgical treatment for carcinoma of the pancreas: experience in 272 patients. *Am.J.Surg.*, **152**, 499–504
16. Warshaw, A. and Swanson, R. (1988) Pancreatic cancer in 1988. Possibilities and probabilities. *Ann.Surg.*, **208**(5), 541–553
17. Spencer, M., Sarr, M. and Nagorney, D. (1990) Radical Pancreatectomy for Pancreatic Cancer in the Elderly: Is it Safe and Justified? *Ann.of Surg.*, **212**(2), 140–143
18. Cohn-Isidore, J. (1991) How I Do it: Editorial Comment. *Amer.J.of Surg.*, **161**(4), 482
19. Grace, P., Pitt, H. and Longmire, W. (1990) Reviews: Pylorus preserving pancreatoduodenectomy: an overview. *Brit.J. of Surg.*, **77**(9), 968–974
20. Haller, D. (1988) Chemotherapy in Gastrointestingal Malignancies. *Semin.Oncol.*, 58–63
21. Longmire, W.P. and Traverso, L. (1981) The whipple procedure and other standard operative approaches to pancreas cancer. *Ca*, **47**, 1706–1711
22. Trede, M. and Gunther, S. (1988) The Complications of Pancreatectomy. *Ann.Surg.*, **207**(1), 39–47
23. Connolly, M., Dawson, P. and Michelassi, F. (1987) Survival in 1001 patients with carcinoma of the pancreas. *Ann.Surg.*, **206**, 366–373
24. Joensuu, H., Alanen-Kalle, A. and Klemi-Pekka, J. (1989) Doubts on "Curative" Resection of Pancreatic Cancer. *Lancet* **1**, 953–954
25. Klijn, J.G.M., Setyono-Han, B., Bakker, G.H., Henkelman, M.S., Portanger, H. and Foekens, J.A. (1987) Effects of Somatostatin Analong (Sandostatin) Treatment in Experimental and Human Cancer. In: *Hormonal Manipulation of Cancer: Peptides, Growth Factors, and New (Anti) Steroidal Agents*, ed: Klijn, J.G.M., New York; Raven Press, pp. 459–468
26. Weger, A., Glaster, K., Schwab, G., Oefner, D., Bodner, E., Auer, G. and Mikuz, G. (1991) Quantitative nuclear DNA content in fine needle aspirates of pancreas cancer. *Gut*, **32**(3), 325–328

*(Accepted by S. Bengmark 21 January 1993)*

### INVITED COMMENTARY

The prognosis of pancreatic cancer is so poor that any attempt to present data on the factors that influence survival is welcome. Dr Brower and colleagues show that certain histological features of the tumour affect long-term survival, although this discovery is not entirely new.

The authors report a good correlation between prognosis and three histopathological factors: extent of local growth, state of regional lymph nodes and degree of cellular differentiation. Tumour size did not accurately predict prognosis, although patients with bulky tumours did badly because these were generally associated with local invasion and lymph node metastasis.

Previous workers have considered this problem. Tumour size alone does not appear to affect prognosis[1]. Small tumours (< 2cm) can still present with advanced local disease or distant spread at the time of diagnosis. In one series 71 per cent of patients with carcinomas < 2cm had already reached stage II-IV by the time of pancreatic resection[2] and in another series 56%[3]. Nevertheless, small cancers generally carry higher rates of resectability and better survival[4-6], possibly because they are diagnosed at an earlier stage or are less biologically aggressive.

This histological characteristics of the resection specimen have also been the object of study. Malignant infiltration of the pancreatic capsule, proximity of the tumour to lymphatic or blood vessels, round cell infiltration at the tumour margin and epithelial atypia in the pancreatic ducts away from the tumour are all factors that have been associated with a poor prognosis[7]. The location of a tumour in the pancreatic body and tail or an eccentric site within the head (far from the duct) are also unfavourable factors[8]. To obtain these data, endoscopic ultrasonography can make a valuable contribution to preoperative staging[9], supplementing conventional abdominal ultrasound and CT Scan. Another crucial factor in determining the outcome is the presence of tumour-free margins in the resected part[10,11].

Regional lymph node involvement has received a good deal of attention. As Brower and colleagues report, lymph node metastasis usually conveys a very poor prognosis[3,12-14], although other reports do not confirm such a clear correlation[1,10]. Likewise, poorly differentiated tumours generally do worse, although some studies have not found this feature relevant[15].

All these histopathological factors reflect the tumour's biological aggressiveness, and this may better be evaluated using modern techniques such as analysis of nuclear DNA content. Some studies of pancreatic cancer have observed a good correlation between resectability/survival and certain characteristics of nuclear DNA[16,17]. Thus cancers with tumour stemlines in the diploid region and with fewer than 6% S phase cells have a more favourable prognosis. Quantitative studies of DNA content may well provide further important information for planning treatment.

### *References*

1. Connolly, M.M., Dawson, P.J., Michelassi, F., Moossa, A.R. and Lowenstein, F. (1987) Survival in 1001 patients with carcinoma of the pancreas. *Ann. Surg.*, **206**, 366–373
2. Manabe, T., Miyashita, T., Ohshio, G., Nonaka, A., Suzuki, T., Endo, K., Takahashi, M. and Tobe, T. (1988) Small carcinoma of the pancreas. Clinical and pathologic evaluation of 17 patients. *Cancer*, **62**, 135–141

3. Tsuchiya, R., Takatoshi, N., Harada, N., Miyamoto, T., Tomioka, T., Yamamoto, K., Yamaguchi, T., Izawa, K., Tsunoda, T., Yoshino, R. and Eto, T. (1986) Collective review of small carcinomas of the pancreas. *Ann. Surg.*, **203**, 77–81
4. Nix, G.A.J.J., Schmitz, P.I.M., Wilson, J.H.P., Van Blankenstein, M., Groeneveld, C.M.F. and Hofwijk, R. (1984) Carcinoma of the head of the pancreas. Therapeutic implications of endoscopic retrograde cholangiopancreatography findings. *Gastroenterology*, **87**, 37–43
5. Trede, M. (1985) The surgical treatment of pancreatic carcinoma. *Surgery*, **97**, 28–35
6. Carter, D.C. (1990) Cancer of the pancreas. *Gut*, **31**, 494–496
7. Mannell, A., Van Heerden, J.A., Weiland, L.H. and Ilstrup, D.M. (1986) Factors influencing survival after resection for ductal adenocarcinoma of the pancreas. *Ann. Surg.*, **203**, 403–407
8. Nix, G.A.J.J., Dubbelman, C., Srivastava, E.D., Wilson, J.H.P., Boender, J. and De Jongh, F.E. (1991) Prognostic implications of the localization of carcinoma in the head of the pancreas. *Am. J. Gastroenterol.*, **86**, 1027–1032
9. Rösch, T., Braig, C., Gain, T., Feuerbach, S., Siewert, J.R., Schusdziarra, V. and Classen, M. (1992) Staging of pancreatic and ampullary carcinoma by endoscopic ultrasonography. Comparison with conventional sonography, computed tomography, and angiography. *Gastroenterology*, **102**, 188–199
10. Lygidakis, N.J., Brummelkamp, W.H. and Tytgat, G.N.J. (1986) Periampullary and pancreatic head carcinoma: facts and factors influencing mortality, survival and quality of post-operative life. *Am. J. Gastroenterol.*, **82**, 968–974
11. Trede, M., Schwall, G. and Saeger, H.S. (1990) Survival after pancreatoduodenectomy. 118 consecutive resection without an operative mortality. *Ann. Surg.*, **211**, 447–458
12. Cameron, J.L., Crist, D.W., Sitzmann, J.V., Hruban, R.H., Boitnott, J.K., Seidler, A.J. and Coleman, J.A. (1991) Factors influencing survival after pancreatoduodenectomy for pancreatic cancer. *Am. J. Surg.*, **161**, 120–125
13. Crist, D.W., Sitzmann, J.V. and Cameron, J.L. (1987) Improved hospital morbidity, mortality and survival after the Whipple procedure. *Ann. Surg.*, **206**, 358–365
14. Nagai, H., Kuroda, A. and Morioka, Y. (1986) Lymphatic and local spread of T1 and T2 pancreatic cancer. A study of autopsy material. *Ann. Surg.*, **204**, 65–71
15. Kalser, M.H., Barkin, J. and MacIntyre, J.M. (1985) Pancreatic cancer. Assessment of prognosis by clinical presentation. *Cancer*, **56**, 397–402
16. Joensuu, H., Alanen, K.A. and Klemi, P.J. (1989) Doubts on "curative" resection of pancreatic cancer. *Lancet*, April, **29**, 953–954
17. Weger, A.R., Glaser, K.S., Schwab, G., Oefner, D., Bodner, E., Auer, G.U. and Mikuz, G. (1991) Quantitative nuclear DNA content in fine needle aspirates of pancreatic cancer. *Gut*, **32**, 325–328

